# Revisiting child and adolescent health in the context of the Sustainable Development Goals

**DOI:** 10.1371/journal.pmed.1003449

**Published:** 2020-10-30

**Authors:** Zulfiqar A. Bhutta, Kathryn M. Yount, Quique Bassat, Artur A. Arikainen

**Affiliations:** 1 Centre for Global Child, The Hospital for Sick Children, Toronto, Canada; 2 Institute for Global Health & Development, The Aga Khan University South-Central Asia, East Africa & London, United Kingdom; 3 Emory University, Atlanta, Georgia, United States of America; 4 Barcelona Institute for Global Health, Barcelona, Spain; 5 Public Library of Science, Cambridge, United Kingdom and San Francisco, California, United States of America

The year 2020—five years since 189 countries signed the Sustainable Development Goals (SDGs)—has been consumed by the global response to COVID-19. To date, this pandemic has resulted in over 38 million cases and well over a million deaths [1]. One collateral effect of COVID-19 has been the setting aside of many SDGs and efforts to track progress towards them. Attention to children during the pandemic has concentrated on school closures, food insecurity, and access to care within health systems taxed by COVID-19 mitigation and response efforts [2]. The situation of child and adolescent health before COVID-19, and consequences of the pandemic on specific health targets for SDG 3, therefore deserve attention.

As the Millennium Development Goals (MDGs) ended in 2015, activities and plans addressed the global compacts for reducing child mortality. A focus was on determinants, such as maternal and child undernutrition, gender inequities, and intersecting vulnerabilities. Because adolescents were largely ignored in the MDG process, advocacy and effort were invested to make them central to the SDG agenda. The renewed global strategy for Every Woman Every Child, launched by the UN Secretary General in 2013, was a segue to the SDGs and an effort to go beyond survival toward a transformative agenda that included healthy development [3]. Advocacy for the integration of health, nutrition, and early child development led to the development of the nurturing care framework [4].

As we examine the situation more than 5 years into the SDGs, several concerns emerge. Despite progress, the field remains fragmented, with limited actions in countries to develop integrated strategies for reproductive, maternal, newborn and child health (RMNCH), or inclusion of adolescent health within national plans [5]. Work on the drivers of adolescent health, well being, and empowerment is underway but has yet to translate into a reasonable global strategy. This lag stems from complex, multilevel social influences during adolescence [6], insufficient disaggregation of data on adolescents, suboptimal measurement and a lack of well-defined indicators [7–9], and limited evidence on the differential impacts of social policies and programs [8] within adolescence and between adolescence and adulthood. Within health systems, many nutrition programs remain poorly integrated with other RMNCH programs and few have substantive links with sectors outside health. With the unfinished agenda for maternal, newborn and child deaths, rigorous studies to address mechanisms and hitherto unrecognized causes of excess mortality are just beginning to yield results, albeit with older pediatric age groups remaining significantly understudied, even at the simplest descriptive level [5]. Effort is limited to bring mental-health programming to women and children, especially in conflict settings or emergencies such as the COVID-19 pandemic. These silos in research, planning and policy, and service delivery apply equally to other sectors, and to multi-sectoral planning and implementation at country level. To address the SDGs, we must consider life in the 21^st^ century—including the disruptions of technological change, economic shocks, climate change, and conflict and security.

Thus, as we seek to reposition child health in the context of the SDGs and to eliminate preventable child deaths, we need a life-course perspective that is situated in a broader social and structural context. This framework recognizes the multilevel social contexts into which children are born and the long-term health impacts of early exposure to deprivation, inequality, and unsustainable growth. We recognize that healthy trajectories from birth to adolescence and beyond depend on the extent to which key stakeholders—governments, businesses, and civil societies—prioritize investments in human capabilities, reductions in inequality, and inclusive, sustainable economies [10]. In conceptualizing the life course from birth to adulthood in terms of trajectories of healthy growth, learning, and development, there are predictable touch points where investments can be made and progress monitored—growth before age 2 years, readiness for school, developmental and academic milestones in school, and social milestones with respect to family, peer, and dating relationships. A contextually situated life-course perspective for human development across childhood, as opposed to early childhood alone, conceptualizes exposures and opportunities from conception to adulthood, with each life stage building on the previous stages in social context. This ‘contextual’ and ‘cumulative’ perspective is important for understanding trajectories of survival, health and development throughout childhood and adolescence and the ‘reciprocal’ social and economic contributions that healthy, fulfilled adults can make to inclusive, sustainable societies. Recognizing the inter-connectedness of capabilities also allows investments in one domain to attenuate vulnerabilities in another (e.g., ensuring that all children receive a high-quality education may attenuate vulnerabilities associated with stunting among the marginalized). Given the strong predictions of cumulative disadvantage for long-term health, disrupting these trajectories requires collaboration among key stakeholders.

Some 2 decades after the world embarked on the MDGs, we need to focus on reaching the unreached through cross-sectoral delivery platforms and strategies that prioritize the most excluded and vulnerable [11]. Still, high-quality programs within the health system remain important, and require redesign to meet the needs of children and adolescents. Achieving this high quality will require inclusion of trained providers, competent delivery systems, and an emphasis on dignity and trust in provider–client relationships, especially with the most excluded. Today, the quality of services for sick children is substandard, with only 40% of recommended Integrated Management of Childhood Illness (IMCI) clinical interventions delivered in a visit. System-wide deficits include minimal service offerings for adolescents, or even children beyond the age of 5 years, and misplaced services, with many births occurring in facilities that cannot care for the very sick newborn. The path forward for improvement is to focus on structural solutions that place the child and family at the center of the health system’s mission, address social inclusion of marginalised groups, and help develop high-quality health systems for improving child health [12]. Health can be an entry point, yet collaboration across stakeholders and sectors, such as education, social media, and social protection, are critical to reduce disparities and foster capabilities for health and well-being.

The SDGs have a broader agenda, translated into “survive-thrive-transform” in the Global Strategy for Women’s, Children’s and Adolescents’ Health 2016–2030, with major targets and indicators in the health sector and beyond. We remain concerned that the gains in early child health painstakingly achieved in the MDG period are at risk of slowing down and losing priority. The global challenges of improving survival and health from birth through adolescence remain, and the world needs to redouble its efforts to do better, rather than declare victory prematurely and move on.

In a forthcoming *PLOS Medicine* special issue [13], we are inviting impactful research in this important area on strategies to monitor and combat child mortality globally from birth through adolescence, school-age health and welfare, marginalised populations, and the environmental impacts on children’s health. We hope that this special issue will help to redirect attention to child and adolescent health in years to come.
